# Academies’ Call to Action: Air Pollution Threatens Global Health

**DOI:** 10.5334/aogh.2660

**Published:** 2019-12-16

**Authors:** Marcia McNutt, Victor Dzau

**Affiliations:** 1 US National Academy of Sciences, US; 2 US National Academy of Medicine, US

## Abstract

Policies and actions that promote clean air have been shown to provide a triple benefit – to human health, to the environment, and to the economy. Five national academies are issuing an urgent call for all stakeholders to take immediate action to combat this global threat to human health and sustainable development.

Air pollution is a shared by-product of more than a century of industrialization that impacts all nations in all hemispheres. No part of our globe has escaped its impact, no matter how remote or uninhabited. In this issue of *Annals of Global Health*, five National Academies of Sciences and Medicine from South Africa, Brazil, Germany and the United States of America have joined forces to issue an urgent call for a new global compact for governments, businesses and citizens to reduce air pollution in all countries. The statement from the academies is intended to provide further input for the global climate action summit to be convened by the UN Secretary General in September 2019, where air pollution and public health will be an important issue.

This call to action from the academies includes a request for emissions controls in all countries and proper monitoring of key pollutants – especially fine particulate (PM_2.5_) air pollution. PM_2.5_ is composed of the smallest airborne particulates, and these fine particles can enter and impact all organs of the body. Make no mistake: the current controls on PM_2.5_ save lives. The science academies furthermore specify the need for increased funding to tackle the problem and substantial investment in measures to reduce air pollution.

Knowledgeable scientists from these academies have concluded that the scientific evidence is unequivocal: air pollution is responsible for at least 5 million premature deaths per year. The problem has grown rapidly over the past three decades, and absent aggressive intervention, the number of deaths due to ambient air pollution will double by 2050. This burden disproportionately affects the world’s most vulnerable populations. According to the WHO, approximately 91% of premature deaths due to air pollution occurred in low- and middle-income countries, and the greatest number in the WHO South-East Asia and Western Pacific regions. As a result, failure to address air pollution levels risks contributing to global health inequity. Beyond the health impacts, air pollution is responsible for enormous economic losses: an estimated $3.8 trillion (USD) per year. These losses are so great that they threaten sustainable development.

There is no uncertainly concerning the most important source of harmful air pollution: combustion. The specific sources include combustion of coal, oil and gas, as well as combustion of biomass fuels. The insidious nature of air pollution is that it invades our global commons – the very air we breathe. Global wind patterns transport it tens of thousands of kilometers from its source. For that reason, even nations that enact policies and technologies to limit their sources of harmful air pollution are at risk from the emissions of their upwind neighbors.

But there is good news. Air pollution can be controlled, and control is highly cost-effective. Consider the experience of high-income countries such as the United States (US). The US has reduced air pollution by 70% since 1970 while growing our economy by 250%. Many other nations have reported similar cost/benefit. It is estimated that each dollar invested in control of air pollution yields an economic return of $30.

No one academy or nation owns this problem or the solutions. Under the leadership of the German Leopoldina, this core group of academies convened to examine the science, technical, and policy implications of air pollution in a relatively condensed time frame and obtain approval for the statement from their academies through their various quality control procedures. They now invite all academies of the world and other scientific stakeholders to work with their citizens, governments, and industries to address this critical issue, and share a wide range of creative solutions. With our global reach and unparalleled ability to tap into the brightest minds in diverse fields, the five science and medical academies are well positioned to elevate this pressing issue.

The US National Academies of Sciences, Engineering, and Medicine, as the Congressionally-chartered advisors to the nation on matters where evidence can inform better public policy, are rising to the challenge of how to address this problem through the creation of a new program in Environmental Health, a field that examines how the environment impacts human health. Prior to the creation of this new cross-cutting theme area, problems such as air pollution and public health would be difficult to tackle because of the disciplinary nature of most scientific organizations. Monitoring and transport of air pollution would typically be the purview of the atmospheric sciences, which would reside in a geosciences division. A medicine division would have jurisdiction over tracking patterns of public health and connecting cause and effect, determining which emissions are most harmful. Engineers are more likely to own the solution space: what sort of new technologies can prevent or limit harmful pollution, ideally at its source. Social scientists, in yet another division, would have insight as to why certain solutions may or may not be adopted despite efficacy. Bringing all of these elements together within the same program with the goal of addressing those environmental factors that impact public health will reduce the organizational and other barriers to the problem.

The US National Academies’ rich history of providing guidance and leadership in environmental health research and policy includes a wide array of work, including consensus reports that have had significant impacts on toxicity testing, risk assessment and problem formulation, and science for the future of the US Environmental Protection Agency. The Academies also have a range of other work in fields that could intersect with environmental health, including building healthy, resilient, and sustainable communities after disasters and characterizing risk in climate change assessments.

In closing, control of air pollution has a number of co-benefits. Prior to the passage of the Clear Air Act in the US (1972), freshmen attending college in Pasadena, California, in September were unaware that there was a towering mountain range to the north until the winter rains cleared the air months later. Today, these beautiful mountains are always visible. Furthermore, controlling air pollution will help slow the pace of global climate change, something we can all celebrate.

